# Cyclodextrins as Emerging Therapeutic Tools in the Treatment of Cholesterol-Associated Vascular and Neurodegenerative Diseases

**DOI:** 10.3390/molecules21121748

**Published:** 2016-12-20

**Authors:** Caroline Coisne, Sébastien Tilloy, Eric Monflier, Daniel Wils, Laurence Fenart, Fabien Gosselet

**Affiliations:** 1 Laboratoire de la barrière hémato-encéphalique (LBHE), University Artois, EA 2465, Lens, F-62300, France; laurence.tilloy@univ-artois.fr; 2 Unité de Catalyse et de Chimie du Solide (UCCS), University Artois, CNRS, UMR 8181, Lens, F-62300, France; sebastien.tilloy@univ-artois.fr (S.T.); eric.monflier@univ-artois.fr (E.M.); 3 ROQUETTE, Nutrition & Health R & D, 62136 Lestrem, France; DANIEL.WILS@roquette.com

**Keywords:** cyclodextrins, cholesterol, atherosclerosis, neurodegenerative diseases, vascular diseases, Niemann-Pick disease type C (NPC), Alzheimer’s disease (AD), blood-brain barrier (BBB), HPβCD, KLEPTOSE^®^ CRYSMEB

## Abstract

Cardiovascular diseases, like atherosclerosis, and neurodegenerative diseases affecting the central nervous system (CNS) are closely linked to alterations of cholesterol metabolism. Therefore, innovative pharmacological approaches aiming at counteracting cholesterol imbalance display promising therapeutic potential. However, these approaches need to take into account the existence of biological barriers such as intestinal and blood-brain barriers which participate in the organ homeostasis and are major defense systems against xenobiotics. Interest in cyclodextrins (CDs) as medicinal agents has increased continuously based on their ability to actively extract lipids from cell membranes and to provide suitable carrier system for drug delivery. Many novel CD derivatives are constantly generated with the objective to improve CD bioavailability, biocompatibility and therapeutic outcomes. Newly designed drug formulation complexes incorporating CDs as drug carriers have demonstrated better efficiency in treating cardiovascular and neurodegenerative diseases. CD-based therapies as cholesterol-sequestrating agent have recently demonstrated promising advances with KLEPTOSE^®^ CRYSMEB in atherosclerosis as well as with the 2-hydroxypropyl-β-cyclodextrin (HPβCD) in clinical trials for Niemann-Pick type C disease. Based on this success, many investigations evaluating the therapeutical beneficial of CDs in Alzheimer’s, Parkinson’s and Huntington’s diseases are currently on-going.

## 1. Introduction

Alteration in lipid metabolism is a feature of atherosclerosis and several neurological disorders (NDs) suggesting that innovative pharmacological approaches aiming at counteracting cholesterol imbalance display promising therapeutic potentials in these diseases. However, targeting cholesterol metabolism is not so easy because first, the brain and the peripheral cholesterol metabolisms are quite different and secondly because the central nervous system (CNS) is isolated from the periphery by the blood-brain barrier (BBB) which impedes drug delivery into the brain. Furthermore, the BBB actively participates in the complex brain cholesterol homeostasis (this metabolism is reviewed in [1]) and contributes in disease pathogenesis and development [2]. The BBB consists in a sophisticated network of complex tight junctions between adjacent endothelial cells associated with low pinocytotic activity and lack of fenestrae preventing the paracellular and transcellular diffusion of hydrophilic molecules across the BBB, respectively [3]. However, in addition to physical barrier properties, the BBB establishes a metabolic barrier allowing permanent exchanges between blood and CNS. These are achieved by means of transmembrane transporters, receptors and cytoplasmic enzymes involved in nutrient transportation across the BBB as well as constant elimination of toxic metabolites from the CNS and exclusion of circulating xenobiotics to enter the CNS processed by ATP-dependent efflux pumps such as ABCB1 (P-glycoprotein (P-gp)) [4] or ABCG2 (breast cancer resistance protein (BCRP)) (for a review see [5]). As a result, CNS disorders still remain very difficult to treat. In such a context, the usage of cyclodextrins and their derivatives appears as a highly relevant therapeutical challenge. These molecules, synthetized by enzymatic cleavage of starch, have extensively been used worldwide in numerous industrial production processes in food, cosmetics, agriculture, environment, medicine and chemistry. In the pharmaceutical area the formulations of an increasing amount of marketed drugs include CDs due to their capacity to enhance drug solubility, drug bioavailability, and chemical stability [6,7,8] in addition to their diminished cell toxicity and the capability to mask the bitterness of many active compounds or adjuvants [9]. This has been related to the physicochemical structure of CDs able to trap drug candidates into their cavity enabling their use in drug delivery to facilitate body tissue penetration of hydrophilic or hydrophobic drugs. In addition CDs can directly interfere with biological membranes, without readily penetrate through, to extract lipids such as cholesterol from cell membranes modifying cell metabolism and functions [10]. 

As atherosclerosis is characterized by an abnormal accumulation of cholesterol in the artery vessel wall upon inflammatory processes, CD-based therapy as a cholesterol-sequestrating agent has recently demonstrated promising advances using some CD derivatives such as KLEPTOSE^®^ CRYSMEB and 2-hydroxypropyl-β-cyclodextrin (HPβCD) [11,12,13]. However, getting access to the brain still remains a major challenging task for pharmaceutical industries in their attempts to improve drug delivery to the brain and to evaluate xenobiotic toxicity. In this context, the therapeutical benefits of CD at the BBB level are highly promising in regards to the ability of CDs to extract lipids from cell membranes in addition to its drug delivery potential. 

## 2. Structural Aspects of CDs

CDs represent a family of cyclic oligosaccharides prepared from starch after enzymatic digestion and transglycosidation. They are shaped as truncated cones with an outer hydrophilic surface of (α 1 → 4)-linked α-d-glucopyranose units and a lipophilic internal cavity [14]. Mostly studied CDs are made up of six, seven or eight glucopyranose units defining the native α, β and γ-CDs, respectively. In addition, the number of glucose units define the diameter of the internal cavity (0.50 nm, 0.62 nm and 0.80 nm for α, β and γ-CDs, respectively [15]). Many derivatives of CDs have been generated in order to improve the water-solubility of these molecules as compared to the natural CDs and more specifically β-CDs which are poorly water-soluble, improving de facto their commercial interest. A frequently used approach consists in partial methylation by substitution of any of the hydroxyl groups. Therefore, many CD derivatives display different degrees of substitution conferring specific biochemical and biological properties to the CDs (Table 1) (for a review see [16]). 

The major physicochemical property of CDs that is common to all of them lies in their capacity to form inclusion complexes with many compounds and more interestingly with drugs. Although all types of CDs can achieve inclusion complexes, the size of their inner cavity represents the main concern as to which CD can display better success in complex formation. To this aim, of the three native CDs, β-CDs have been shown to exhibit the most suitable cavity size for hosting many biological compounds such as hormones, vitamins, and other molecules frequently used in the biology field [17].

The ability of the CDs to form inclusion complex has two main advantages. First it helps solubilizing poorly hydrophobic compounds in using CD as a so-called carrier partner in order to facilitate their aqueous dissolution. Molecules of interest that are hydrophobic can easily get encapsulated within the hydrophobic cavity of the CDs while the hydrophilic outside makes the whole complex water-soluble.

The second advantage of inclusion complex formation consists in greatly modifying the properties of the molecule of interest (more precisely “drugs” in our case) in many ways such as improving drug stability, bioavailability, oral administration and drug interaction with biological membranes or cells. This latter advantage can easily explain the reason why CDs have appealed so much attention and have been marketed worldwide in many industry areas from food, cosmetics, environmental engineering to chemical, pharmaceutical production and development. The toxicity profile of the CDs and derivatives has been extensively evaluated [18,19]. When administered orally, CDs are generally considered safe as they do not cross the intestinal barrier [20], however, for the same CD the route of administration can modify its toxicity as demonstrated for native β-CD, which exhibits a limited toxicity after oral administration in animals as the acceptable daily intake has been limited to 5 mg/kg of body weight by the International Program on Chemical Safety (IPCS; WHO Food Additives Series 32; http://www.inchem.org/documents/jecfa/jecmono/v32je13.htm), whereas parenteral or subcutaneous injections at higher doses get nephrotoxic affecting proximal tubules [21,22]. The mode of clearance of CDs from the organisms also depends on the route of administration. For example, HPβCD is mainly eliminated by glomerular filtration in the kidneys and excreted into urine [23] after intravenous injection in rats, whereas oral administration is mainly excreted through faeces in rats and dogs [24].

## 3. Modes of Action of CDs

In the pharmaceutical field, novel CD-based technologies of commercial interest are constantly being developed favoring the biological performances of the CDs mostly in regards to drug delivery, biological safety and therapeutic efficiency. These novel CDs are mainly derived from native β-CD and their properties mainly depend on their degree of substitution (Table 1). These involve the methylated β-cyclodextrin derivatives such as the randomly methylated β-cyclodextrins (RAMEβ and KLEPTOSE^®^ CRYSMEβ displaying 12.6 and four methyl groups, respectively), the HPβCD with hydroxypropyl groups randomly substituted onto the β-cyclodextrin molecule, and also the sulfobutylether-7-β-cyclodextrin (SBE7-β-CD) that are currently evaluated for the treatment of neurodegenerative disorders and atherosclerosis. Additionally, β-CDs have proven to be very useful in therapy as they have not shown any hypersensitivity reaction, unlike Sugammadex. This modified γCD used in anesthesia to reverse the effect of neurovascular blocking drugs has been involved in allergic response in some patients [25,26]. As therapeutic agents, the mode of action of CDs and their derivatives can occur in two ways. The first one implies the direct biological action of the CDs on cell membranes whereas the second one is rather indirect in using the encapsulation potentiality of CDs as drug carriers.

### 3.1. Direct Action of the CDs on Cell Membranes

**Figure 1:**
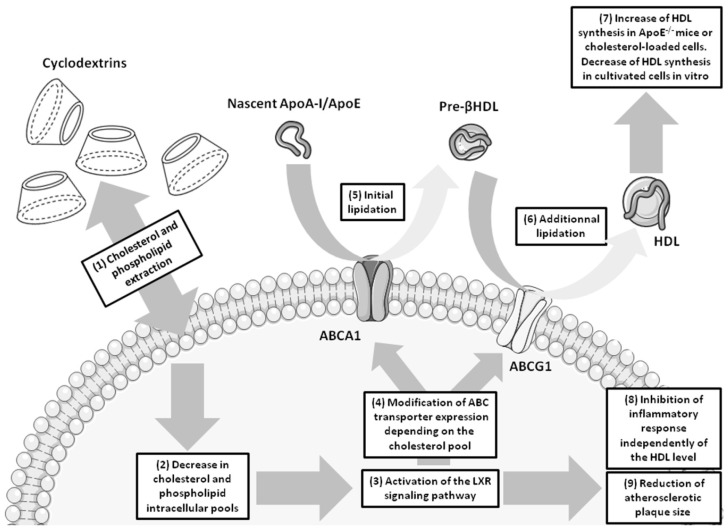
Impact of β-CDs on reverse cholesterol transfer (RCT) and in atherosclerosis. As showed in Table 2, β-CD family shows promising therapeutic properties in atherosclerosis field. β-CDs are able to extract cholesterol and phospholipids from cell membrane (1) resulting in the decrease of lipid intracellular pools (2). This process leads to an activation of the liver X signaling (LXR) pathway (3) which in turn regulates the expression of ABCA1 and ABCG1 (4). As a consequence, the formation and lipidation of HDL particles are modified (5 and 6). In vitro, lipid extraction by CDs provokes a down-regulation of ABCA1 and ABCG1 expression and a decrease in the free cholesterol transfer to ApoA-I [12,13]. However, in tissues from ApoE^−/−^ animals or when cells are cholesterol-loaded in vitro, lipid extraction induces an increase in the cholesterol efflux mediated by the up-regulation of these ABC transporters [13] (7). Noteworthy, in brain, apolipoprotein E remains the main cholesterol acceptor forming HDL. CD-mediated activation of LXR pathway inhibits inflammatory response (8) [11,13]. In addition, CDs promote atherosclerotic size plaque reduction in vivo (9) by a HDL-independent mechanism which remains unclear [11,13]. HDL: High density lipoproteins; LXR: Liver X receptor; ApoA-I/ApoE: Apolipoproteins A-I and E.

The direct action of the CDs on cells consists in extracting lipids (cholesterol and phospholipids) as well as some proteins from cell membranes modifying the molecular composition of the lipid bilayers and thus their properties [27] (Figure 1).

Such an observed extraction phenomenon has been described to depend on the cell surface distribution of the CDs at least for the cholesterol whose membrane extraction is preferentially achieved by β-CDs [28,29,30]. This suggests a passive diffusion of the CDs into the lipid bilayer by directing their opened secondary rim towards the lipidic polar groups while remaining anchored to the glycerol-ester groups and phosphate by hydrogen bond formation. In barrier-forming epithelial cells, the effect of CDs has been evaluated in vitro and in vivo in the dermis, in the nasal cavities and in the gut where it promotes drug absorption across these barriers as a result of their cell membrane cholesterol extraction [20,31,32,33]. At the neurovascular endothelial level, the action of CDs toward the BBB endothelium has been studied by Monnaert et al. (2004 [34]) on an in vitro model of the bovine BBB where they described that α-CD removes phospholipids, β-CD extracts phospholipids and cholesterol whereas γ-CD is less lipid-selective than other CDs. It is likely that this cholesterol-depletion deeply affects the cellular process that controls the transfer of membrane cholesterol to extracellular high density lipoproteins (HDL) via the reverse cholesterol transport (RCT, Figure 1). This major pathway regulating cellular cholesterol pools by promoting the cellular cholesterol efflux is mediated by three proteins mostly expressed by all cell types: two of them are ATP-binding cassette transporter family members, ABCA1 and ABCG1 (the first member of the sub-family A and the first member of the sub-family G, respectively) [35], and the scavenger receptor class B member 1 (SR-BI) [36]. The latter mediates bidirectional cholesterol exchanges between cell membrane and HDL whereas ABCA1 interacts with ApoA-I forming a partially lipidated discoidal complex. This complex interacts subsequently with ABCG1 to get additional cholesterol molecules and generates HDL [37]. Because the BBB regulates the complex brain cholesterol homeostasis [38,39], it remains of interest to investigate how CD treatment may impact BBB cholesterol-depletion which in turn may influence ABCA1/G1 expression on brain cholesterol transport and synthesis. When toxicity was assessed, β-CD family (native, HPβCD and RAMEβ) was found more toxic than γ-CD family (native, HPγCD and RAMEγ) and less toxic than α-CD family (native, HPαCD and RAMEα) [34]. More recently, the cytotoxicity of β-CDs, HPβCD and TRIMETHYL-β-CDs has been investigated in a mouse model of immortalized cerebellum microvascular endothelial cells (cEND) and viability testing indicated the non-toxic effect of HPβCD as compared to the toxic β-CDs and TRIMETHYL-β-CDs [40].

In peripheral vascular beds, the degree of methylation of β-CDs has recently been reported to be of prime importance in the ability of the CD to extract cholesterol from cell membranes [12]. This work highlights that the percentage of extracted cholesterol rather depends on the methylation degree of the methylated β-CDs than on cell membrane composition, cellular content or cell type. In this study several β-CD derivatives have been compared in their ability to extract cholesterol from cell membranes of bovine aortic arch endothelial cells (ABAE) and bovine aorta smooth muscle cells (SMCs). The native β-CD and RAMEβ have been shown to extract more efficiently cholesterol from cell membrane than other methylated β-CDs, extracting cholesterol in lower amount. In addition, the authors have reported a decrease in the RCT correlated with the downregulation of ABCA1 and ABCG1. Consequently cholesterol extraction and its resulting depletion from cell membranes may generate a deeper impact in physiology and pathophysiology of cells, body tissue and organ functions as the loss of membrane cholesterol can directly affect the lipid rafts involved in many cell signaling pathways [41,42,43,44]. The other way CD can directly display its biological action consists in the delivery of drug across biological barriers which normally protect the organs against xenobiotics.

### 3.2. CDs as Drug Delivery Systems

CDs are widely used as drug delivery carrier via nasal mucosae, pulmonary-, ocular-, dermal-, intestinal- and brain-barriers as these molecules improve delivery and bioavalability of hydrophilic, hydrophobic as well as lipophilic drugs. For example, it was described on primary bovine brain capillary endothelial cell monolayers mimicking the BBB in vivo that hydroxypropyl-γ-CD and γ-CD increased the permeability of the lipophilic antitumoral drug doxorubicin (DOX) by inhibiting the P-glycoprotein-induced DOX efflux [45,46]. The same effect was reported before in Caco-2 cells, a human in vitro intestinal barrier model, with dimethyl-β-CD delivering an immunosuppressive compound [47]. The property of CDs to extract cell membrane cholesterol modifying thus lipid raft composition which would in turn change the membrane localization of the P-gp and would result in the loss of this efflux-pump function has been suggested. To verify this hypothesis, the enhanced DOX delivery across the BBB upon CD treatment was investigated by means of the in vitro BBB system mentioned before [45,48] and β-CDs. Co-incubation of either RAMEβ or KLEPTOSE^®^ CRYSMEβ with DOX (which is too big to get encapsulated by these β-CDs, unlike γ-CDs and hydroxypropyl-γ-cyclodextrins) increased DOX transport across the BBB by 2 and 3.7, respectively, suggesting an impaired P-gp activity due to lipid raft disorganization [48]. Gil and colleagues (2009, 2012) developed β-CD-based nanocarriers, namely quaternary ammonium β-CD (QA-CD) [49] and β-CD-poly(β-aminoester) [50] nanoparticles, that enhanced drug transport across the intact BBB endothelium. Nanoparticles display the ability to enter cells via endogenous endocytosis and transcytosis pathways based on their small size. In their 2009 study, Gil and colleagues investigated the effect of CD-based nanoparticles (at concentrations up to 500 µg/mL) on the delivery of DOX across bovine brain microvascular endothelial cell monolayers [49]. They did not observe any deleterious effect of the nanocarrier on transendothelial electrical resistance, Lucifer yellow permeability, tight junction and P-gp expressions as well as on cholesterol extraction as compared to controls. In parallel, DOX permeability across the BBB endothelium, probably through endocytic pathway, almost doubled while incorporated in CD-based nanoparticles. In 2012, the same group evaluated the permeability of biodegradable polymeric nanoparticles composed of β-cyclodextrin and poly(β-aminoester) segments across human and bovine brain microvascular endothelial cells [50]. In addition to maintain the integrity of the in vitro BBB systems, these nanoparticles displayed much higher permeability than controls and their capacity to release DOX lasted for almost a month.

**Figure 2:**
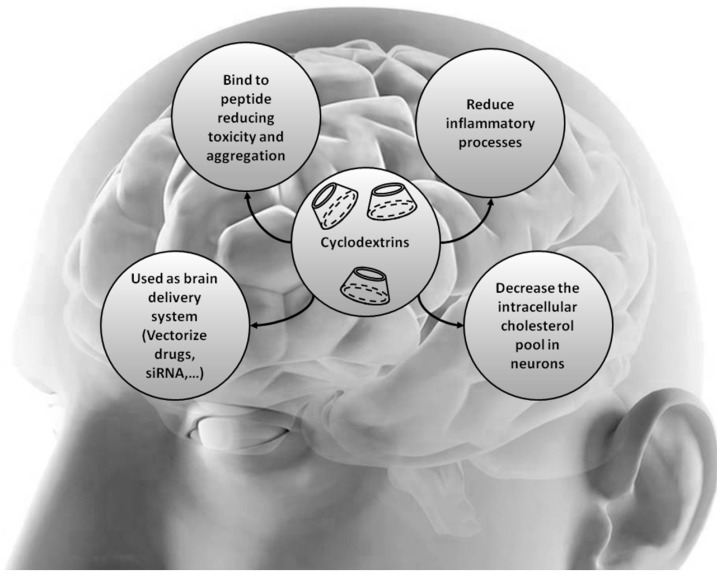
Molecular mechanisms mediated by β-CDs in Alzheimer’s, Parkinson’s, Hugtington’s, Niemann-Pick Type C diseases. Niemann-Pick Type C disease is characterized by intracellular accumulation of unesterified cholesterol and glycosphingolipids. Alzheimer’s, Parkinson’s and Huntington’s diseases are charaterized by abnormal aggregation of peptides leading to the neurodegenerative processes. β-CD family acts at several levels and shows beneficial effects in these neurodegenerative diseases.

CDs have also been extensively used to ameliorate biocompatibility and enhanced bioavailability when incorporated into complexes with active drug compounds, thus enhancing drug efficacy. This will be more extensively discussed later in this review for atherosclerosis and neurodegenerative diseases such as Alzheimer’s and Parkinson’s diseases (Figure 2). In addition, CDs can be used as a carrier enabling the selective binding to biomolecules of interest, as reported for example for cholesterol crystal detection in atherosclerosis ([51], later discussed in this review).

Therefore, both direct and indirect action of the CDs have proven effects on cells and many research approaches constantly evaluate their potential for therapeutic uses for treating many disorders including neurodegenerative and atherosclerosis related-pathologies (summarized in Table 2) (Figure 2).

## 4. Cyclodextrins and Atherosclerosis

The abnormal accumulation of cholesterol into the vessel wall of arteries during atherosclerotic plaque formation represents the major culprit for the vascular alteration observed in atherosclerosis. Inflammatory processes start the recruitment of lipid-laden macrophages into the artery wall that will later form foam cells containing high amount of cholesterol esters [91]. In addition other cell types from the artery wall such as smooth muscle cells (SMCs) and endothelial cells can accumulate cholesterol and get converted into lipid-laden foam cells [92]. This creates an imbalance between excessive cholesterol accumulation and baseline cholesterol elimination in these cells [93] modifying the regulation of cellular cholesterol pools. This regulation in resting conditions is mainly performed by the RCT which involves ABCA1, ABCG1 and SR-B1 transporters that support the efflux of cholesterol from the cell membranes towards the extracellular HDL [36] as previously described in this review (Figure 1). The alteration of the expressions of these three transporters has been reported in the three different cell types forming foam cells in atherosclerotic arteries. However discrepant data were reported from in vitro studies performed with SMCs and macrophages as well as in ex vivo investigations on arteries exhibiting different size of atherosclerotic plaque formation and disease progression. Some studies revealed increased expressions of ABCA1 and ABCG1 in arteries whereas in others ABCA1, ABCG1 and SR-B1 expressions were diminished [94,95,96,97,98].

In this regard, the therapeutical approaches aiming either at preventing atherosclerotic plaque formation, or at stopping the progression of plaque formation or even at reducing already installed atherosclerotic plaques based on CDs’ behavior as cholesterol-sequestrating agents, attempting to solubilize cholesterol within atherosclerotic plaques enhancing thus its clearance and catabolism, have already started to produce promising results. In this line of considerations, Montecucco and colleagues (2015 [11]) highlighted the potential of the KLEPTOSE^®^ CRYSMEβ to treat atherosclerosis in vivo. ApoE-deficient mice fed with either normal or rich-cholesterol diet were intraperitoneally injected either with vehicle (PBS) or with KLEPTOSE^®^ CRYSMEβ (40 mg/kg in PBS/ 3 times a week). CD-stimulated mice, independently of their food diet, showed an increase in HDL-cholesterol and a decrease in triglyceride blood levels but no change in total serum cholesterol and LDL-cholesterol levels in blood compared to their untreated littermates. In addition, KLEPTOSE^®^ CRYSMEβ treatment reduced atherosclerotic plaque size by decreasing the cholesterol accumulation within these lesions when compared to vehicle-control injected littermates.

Inflammatory response was also investigated in this study and authors focused on the associated Th1-mediated immune response. They demonstrated that T lymphocyte content was reduced in atherosclerotic plaques, without any effect on any other cellular or molecular component present in the atherosclerotic plaque such as macrophages, neutrophils, MMP-9 and collagen. In this study the efficiency of KLEPTOSE^®^ CRYSMEβ was more striking in animals receiving the cholesterol-enriched diet than in mice fed with the normal food. This may suggest a better efficacy of this particular CD in the advanced disease in which inflammatory processes are longer settled and high amount of cholesterol are found in the blood circulation. Moreover the mode of action of KLEPTOSE^®^ CRYSMEβ in modulating immune response and Th1 polarization remains to be utterly elucidated. However, the authors omitted to investigate the impact of KLEPTOSE^®^ CRYSMEβ on other cell types forming atherosclerotic plaques such as SMCs and aortic endothelial cells. Recent in vitro studies by us (2016 [12]) focused on the effect of several β-CDs and their methylated derivatives, including KLEPTOSE^®^ CRYSMEB, on the regulation of the cholesterol metabolism (cholesterol accumulation, RCT and HDL formation) in bovine arterial SMCs and ABAE cells. This study demonstrates that β-CDs were efficient in extracting cell membrane cholesterol in both cell types resulting in a reduction of the whole cellular cholesterol content. This has been correlated with the major decreased expression of ABCA1 and ABCG1, but not SR-BI expression, and to the reduction of the cholesterol release from arterial SMCs and aortic endothelial cells.

Another promising CD-derivative candidate able to solubilize cholesterol is the FDA-approved HPβCD. This hydroxypropyl derivative of β-CD displays improved water solubility properties enabling to increase solubilization rate, encapsulation and better delivery of lipophilic drugs [99,100]. In the atherosclerosis context, the evaluation of the effect of HPβCD in vitro has been restricted to macrophages and macrophage-derived foam cells, in which HPβCD stimulate their cholesterol efflux [53,54,55]. Zimmer and colleagues (2016 [13]) have recently assessed the effect of HPβCD in vivo. Using animal model of atherosclerosis (ApoE-deficient mice), the authors reported that ApoE-deficient mice fed with a cholesterol-enriched diet and receiving concomitant subcutaneous injections of the HPβCD displayed a reduced inflammatory response during atherogenesis as ROS and Th1 pro-inflammatory chemokine productions were both diminished compared to control littermates. The plasmatic cholesterol levels from treated mice remained unchanged, however, these mice displayed reduced atherogenesis features such as lower atherosclerotic lesion formation in the aorta as well as reduced amounts of cholesterol crystals (CCs) within the atherosclerotic plaques diminishing thus mechanical damage to biological membranes compared to untreated mice. While tested during on-going atherosclerosis, it promotes regression of established atherosclerotic plaques as well as the solubilization of CCs into water-soluble 27-hydroxycholesterol compared to control animals. The underlying mechanisms of action of HPβCD in vivo have been demonstrated to involve the LXR-mediated signaling pathway for the both anti-atherosclerotic and anti-inflammatory effects observed of HPβCD. As LXR signaling pathway targets genes implicated in the RCT, cholesterol efflux was increased in cholesterol-loaded murine macrophages via upregulation of ABCA1 and ABCG1 expression levels. In human carotid biopsies exhibiting atherosclerotic plaques, HPβCD also promoted the efflux of cholesterol from plaques to supernatants involving as well cholesterol metabolism and LXR-related gene expression profile. HPβCD has been therefore newly considered as an “inverter”, a novel class of pharmaceutics able to reverse the normal function of a biomolecule in order to act the opposite way, here in converting cholesterol into 27-hydroxycholesterol which reduces atherosclerosis [56]. It has to be noticed that LXR signaling pathway has been targeted with significant success in former studies, however, LXR agonists such as T0901317 and GW3965, induced nephrotoxicity associated with lipogenic effects [101,102,103]. As a drug carrier in atherosclerosis treatment, the cellular cholesterol efflux enhancing peptide (CEEP) has been associated to a molecular complex composed of β-CD and chitosan, a cationic polysaccharide adding additional biocompatibility and biodegradability to the complex. In vitro in HeLa cells, β-CD/chitosan complexes exhibited an enhanced capacity to efflux cholesterol from cell membranes as compared to β-CD alone and the addition of CEEP to the β-cyclodextrin/chitosan complex double this efficacy of cholesterol extraction from cell membranes [52]. In 2012, Li and colleagues [51] conjugated carboxy-methyl-β-CD with superparamagnetic ion oxyde nanoparticles exhibiting a selective binding to CCs with the aim to further use as a detection tool in MRI for atherosclerosis.

## 5. Cyclodextrins and Cholesterol-Associated Neurodegenerative Diseases

Some neurological disorders display impaired cholesterol trafficking. The most commonly investigated ones are the abnormal cholesterol storage disease Niemann-Pick type C (NPC) in addition to Alzheimer’s (AD), Huntington’s (HD) and Parkinson’s diseases (PD).

### 5.1. Niemann-Pick type C (NPC)

Niemann–Pick type C (NPC) disorder, known as “childhood Alzheimer’s”, is characterized by an abnormal storage of unesterified cholesterol within the lysosomal compartment in cells with an estimated incidence of 1 in 120,000. This multiorgan affecting disease is due to an autosomal recessive genetic loss of function impacting genes encoding NPC1 (transmembrane protein for 95% of NPC patients) or NPC2 (soluble luminal protein NPC2, for 5% of NPC patients) [104]. These two proteins are involved in the egress of cholesterol from late endosomal/lysosomal (LE/LY) compartments. In NPC patients, principally young children, cholesterol accumulates within cells causing multiorgan failures (liver, lungs), neurodegeneration, seizures, impaired cognition and motor functions leading to early patient death [105]. The discovery of the genes involved in the human pathogenesis, as well as animal models harboring spontaneous mutations or induced mutation in *Npc* genes, greatly contributed to advances in understanding NPC development [106,107,108]. 

The HPβCD was first used as a carrier associated with allopregnanolone, a neurosteroid whose endogenous levels are reduced in *Npc1* deficient mouse, that displays benefit in neuronal cell survival and differentiation [58]. While administered in young mice (7 day-old *Npc1^−/−^* mice), significant health improvements were observed including delayed degeneration and the life expectation of the animals was almost doubled [58,59,60]. Liu and colleagues (2009 [61]) reproduced equal observations when HPβCD alone was injected in 7-day-old *Npc1^−/−^* mice. This supports the direct and solely beneficial effects of HPβCD in treating NPC mouse models. However, when HPβCD treatment was applied in older mice the positive effects of CD on life duration and neuronal function recovery were progressively decreased (older than 7 day-old mice) to be completely lost (almost 2 month-old), whereas it still favors the excretion of sequestered cholesterol as bile acid by the liver. As well the benefits on liver functions and cholesterol load were still observed [62,63]. The outcomes in older animals suggest that the CD was not able to efficiently cross the mature BBB to get a direct access to HPβCD neuronal targets. This is in the same line with in vitro data reported by Monnaert et al. (2004 [34]) in a primary bovine model of the BBB, in which HPβCD crossed the BBB-forming endothelium at a very low rate as compared to other β-CD family members (6.6% ± 0.1% versus 9.3% ± 0.5% for methyl-β-CD and 26.7% ± 1.2% for β-CD). However, one cannot exclude that CDs might act at the BBB level regulating in turn the brain cholesterol metabolism [109]. In order to facilitate the CD delivery directly into the CNS, HPβCD was successfully infused intracerebroventricularly in *Npc1*-deficient mice [64].

The beneficial effects of HPβCD in NPC models remains unclear and have been studied in macrophages in which HPβCD exhibits its cholesterol extraction capacity for relatively high concentration of the CD (from >10 mM to 100 mM), whereas at much lower concentration (<1 mM) HPβCD rather behaves as a cholesterol transferring system between membranes of the LE/LY compartment [54,65], in a similar manner to NPC2 function [110]. Another study using another β-CD derivative supports the fact that β-CD family acts on unesterified cholesterol pool in NPC models. Indeed, the octaarginine-appended β-cyclodextrin (R8-β-CD) has recently been tested in vitro in cells where cholesterol gets accumulated into CHO cells through the impairment of the NPC1 function. R8-β-CD displayed no cell toxicity, was endocytosed within the cell via its peptide moiety (R8) and was able to efficiently reduce intracellular cholesterol pool [57].

Ten years after the discovery of the first benefit of HPβCD in reducing hepatic cholesterol levels although with minimal effect on delaying neurogenesis onset in *Npc1*-null mice due to the non-permeation of the BBB by the CDs [111], HPβCD received orphan drug designation in the US and the EU enabling the start for clinical trials. A Phase I pharmacokinetic trial was completed in children at risk for fungal infections receiving single dose of itraconazole associated with HPβCD [68]. The FDA has approved the administration of HPβCD in twin girls suffering from NPC1. Based on these studies, the National Institutes of Health (NIH) has planned to conduct more HPβCD trials. Actually, a Phase I clinical trial evaluating the efficacy and determining the proper CD dose is ongoing (https://clinicaltrials.gov: identifier: NCT01747135), a phase II and III is currently recruiting patients with neurologic manifestations of NPC1 (NCT02534844) and another one is planned with another HPβCD substitute (NCT02912793). Even though universally used in drug formulations and considered safe [20], HPβCD has displayed effective ototoxicity as reported in the feline model of NPC1 disease in which cochlear outer hair cells were affected by HPβCD treatment [66]. Therefore, investigations about developing safer alternates to HPβCD in modifying its degree of substitution are currently occurring as well as an ongoing clinical trial to evaluate their efficacy in NPC1 treatment avoiding at the same time the side-effect of hearing loss [67]. 

Based on the success of β-CDs in NPC1 therapy, other neurodegenerative disorders exhibiting impaired cholesterol metabolism represent promising target candidates for testing the efficacy of CDs. Besides, the potentiality of CD as drug carrier or stabilizing agent has extensively been employed these last years in drug delivery (Figure 2).

### 5.2. Alzheimer’s Disease

Alzheimer’s disease (AD) is characterized by the cerebral accumulation of β-amyloid peptides (Aβ) [112,113] and numbers of evidence have linked impaired cholesterol metabolism to the pathogenesis of Alzheimer’s disease [1,114,115,116]. Hypercholesterolemia has been reported to be a major risk factor for AD in epidemiologic studies and it even accelerated disease development in a mouse model of AD [117,118]. Moreover, cholesterol and plasma membrane lipid contents have been shown to control Aβ production by interfering with enzyme activity [116]. The use of cholesterol-lowering medication such as statins was able to reduce Aβ production in an AD mouse model [118] and AD progression in patients [119]. High doses of simvastatin reduced the cerebral levels of Aβ_1-40_ and Aβ_1-42_ in the cerebrospinal fluid and brain of guinea pig [120]. In addition, Xiong et al. (2008 [121]) observed that AD patients display higher cholesterol retention in brain. 

Therefore, activation of LXR signaling pathway controlling the cholesterol metabolism, in preventing Aβ deposition has been investigated as therapeutic approach by means of LXR-agonist treatment in the APP23 mice as a mouse model of AD [122,123]. In these studies the LXR-agonist T0901317 was able to cross the BBB in vivo and reduce Aβ burdens in mouse brain tissue, however spatial learning only showed little improvement [123]. This was correlated with an increased ABCA1 expression in astrocytes [123] and in neuronal cells [122]. The key role played by ABCA1 in the pathogenesis of AD was reported earlier by Koldamova and colleagues (2005 [124]) who demonstrated that APP23 mice lacking *abca1* exhibit increased amyloid deposition compared to control animals. Therefore the therapeutic potentials of CDs to treat AD patients are of great relevance. Additionally, β-CDs have been reported to be able to directly bind Aβ _1-40_ [69,70,71], and substantially decrease its neurotoxic effect in vitro. Another β-CD derivative, the per-6-alkylamino-β-CD, has been shown to interfere with the oligomerization process of Aβ_1-42_ responsible in part for Aβ neurotoxicity [79]. Based on this ability of CD to form a complex with Aβ and aiming at inhibiting amyloid fibril formation, mucoadhesive microspheres made of chitosan or alginate and containing β-CDs (either native β-CD or HPβCD) were developed as drug candidates in a nasal delivery system for brain targeting [72]. In vitro assays in a neuroblastoma cell line and ex vivo assays in sheep nasal mucosa as a mucoadhesion model, revealed primarily the absence of cellular toxicity and the effective mucoadhesion of the CD complexes. Yao et al. (2012 [75]) evaluated the beneficials of the HPβCD treatment in vitro and in vivo in the AD mouse model (Tg19959 mice). They observed in vitro a lowering of the membrane cholesterol content in N2a cells which overexpress the Swedish mutant APP (SwN2a). In vivo, HPβCD was subcutaneously injected for 4 months in Tg19959 mice starting at 7 days of age and the authors report an improvement in spatial learning and memory, crossing the immature BBB. A reduction of β-amyloid deposits in mouse brains was observed as a result of a reduced APP protein cleavage as well as an upregulation of *abca1* and *npc1* gene expressions. Via ABCA1 modulation, HPβCD directly acts on increasing cholesterol transport and Aβ clearance. HPβCD treatment has also been reported to increase lifespan and to attenuate behavioral and pathological disabilities in ANPC mice, a mouse model generated by an AD mouse model with heterozygous *Npc1*-deficient mice [116]. 

As drug carriers, CDs have been used to deliver active compounds, alone or incorporated in more complex drug delivery systems, in AD therapeutical approaches. This was the case for tacrine, an acetylcholine-esterase inhibitor approved for the treatment of AD exhibiting low bioavailability. The drug was associated with albumin nanoparticles engulfing CDs (β-CD, HPβCD or SBE7-β-CD) for nose to brain delivery and were successfully tested on ex vivo sheep nasal mucosae exhibiting enhanced mucosal adhesion and permeation abilities compared to albumin nanoparticles alone [73]. Drug delivery to the brain, crossing the BBB, to treat AD was assessed by Cheng et al. (2013) by means of the development of curcumin nanoparticles associated with HPβCD [76]. In this study the curative effect of curcumin was evaluated while HPβCD was solely used for its cryoprotective efficiency. Curcumin was previously reported to bind Aβ preventing Aβ aggregation [125]. The three-month oral administration of the nanoparticles to Tg2576 mice, another mouse model of AD, displayed better cognitive beneficial as curcumin nanoparticles-treated mice were better in memory test outcomes than curcumin treated littermates. However cerebral amyloid plaque density was not reduced using the delivery curcumin complex whereas curcumin alone was able to significantly reduce amyloid plaque burden. The authors explained this latter disappointing observation in regards to memory improvements induced with curcumin nanoparticles due to the fact that amyloid plaque size is barely correlated with memory loss [126,127]. Lately, HPβCD or SBE7-β-CD associated with the neurosteroid allopregnanolone was shown to greatly improve the delivery and neurogenic efficacy of the drug upon diverse modes of administration (subcutaneous, topical, intramuscular and intravenous) in various animal models (rabbits, rats, mice, the triple transgenic Alzheimer’s mouse model 3xTgAD) [77]. Shan and colleagues (2016) recently designed novel spray-dried microspheres prepared with β-CD supplemented with two active compounds: chitosan and phospholipids [74]. Chitosan has been described to inhibit Aβ formation and suppress neurotoxic oxidative stress (for review [128]). Phospholipids, acting as acetylcholine precursor, would counteract the loss of cholinergic neurons responsible for impaired cognition in AD patients. The chitosan/phospholipid/β-CD microspheres were tested in vivo in rats modeling AD following Aβ_25-35_ injection into the hippocampus. In these animals, learning and memory were improved according to Morris water maze test data compared to untreated littermates. The underlying mechanisms rely in the decreased expression, upon chitosan/phospholipid/β-CD microspheres injection, of the protein kinase C-δ, whose overexpression in AD has been linked to NF-κB activation leading to central inflammation [129], and a reduced microglia activation in the rat hippocampus, both taking part in neurodegeneration and cognitive impairment in AD. In the same line of research, Yalcin and colleagues (2016) have evaluated the effect of nasal administration of spray-dried polymeric microspheres made of chitosan or sodium alginate containing HPβCD [78]. They observed that rat hippocampi were protected against Aβ_1-42_-induced neurotoxicity as lipid peroxidation, ROS production and apoptotic signals were diminished after HPβCD microspheres administration. Both study results support the evidence that the association of β-CDs with other therapeutical compounds in microspheres is relevant and promising for AD therapy as applying phospholipids, suppressors of neurotoxic oxidative stress do readily ameliorate cognitive impairment in animal models of Alzheimer’s disease.

### 5.3. Parkinson's Disease (PD)

The aggregation of the α-synuclein protein, as well as its misfolding, have been associated with the development and progression of Parkinson’s disease (PD) [130]. These are the consequences of the alteration of the activity of two intracellular protein degradation systems: ubiquitin-proteasome system and the autophagy-lysosomal pathway. The regulation of the latter system has been correlated with the essential activity of the transcription factor EB (TFEB) [131,132]. Recently, HPβCD has been shown to activate TFEB, which stimulated the autophagy-lysosomal pathway that increased the autophagic clearance of α-synuclein aggregates in a human neuroglioma cell line [83]. These findings emphasize the major role of HPβCD as a promising therapeutical agent for enhancing α-synuclein aggregate degradation in disease progression. A previous study has reported an efficient action of RAMEβ in reducing the aggregation of the α-synuclein protein in a rat neuroblastoma cell line in vitro and in vivo in a transgenic mouse model overexpressing α-synuclein [86], though the underlying mechanisms of action of the CD still remain to be elucidated. Therefore, it remains unclear whether CD-mediated effects on cholesterol metabolism are involved in their promising effects in PD and there is a need for further investigations.

l-DOPA is an amino acid precursor of dopamine that is extensively used for the treatment of PD, however very biologically unstable. l-DOPA loaded CD-based nanosponges have been generated by Trotta et al. (2016 [80]) using molecular imprinting with encouraging results for the further storage and controlled delivery of l-DOPA in PD patients. CDs are also used as drug carriers to promote the delivery of dopamine to the brain by nasal route, in particular SBE7-β-CD [87] or maleic acid solution containing HPβCD [84] have shown promising results.

As previously mentioned here for Aβ aggregation [125], the association of β-CD with curcumin was shown to increase the potency of the curcumin in inhibiting α-synuclein aggregation [81], and in dissolving preformed aggregates [82]. HPβCD conjugated to the agonist of dopamine receptor D3, D-264, enhanced its ability to cross the BBB compared to the parent molecule as observed in PD rat models in vivo and in vitro in the hybridoma dopaminergic MN9D cells [85].

### 5.4. Huntington’s Disease

Huntington’s disease (HD) is a rare autosomal dominant neurodegenerative disease caused by a mutation within the *Huntingtin* gene that induces the expression of a toxic Huntingtin protein (HTT) responsible for the neurodegeneration processes leading to motor, behavioral and cognitive dysfunctions. In addition, findings suggest that altered cholesterol homeostasis may contribute to the pathogenesis of the disease as well as the increased expression of caveolin-1, which displays an important role in cholesterol transport and homeostasis [133]. Aiming at reducing the expression of the mutant *htt* gene by means of RNA (siRNA) interference, Godhino and colleagues (2013) investigated the potential of β-CD derivatives as siRNA neuronal carriers [89]. The authors observed a reduced expression of the *htt* gene in vitro in rat neuronal cell line expressing the human HTT protein and in human fibroblasts. In vivo, repeated brain injections of β-CD/siRNA complexes resulted in promising outcomes such as the decreased HTT expression and the selective alleviation of motor deficits in the brains of R6/2 animals, a mouse model of HD. The hydrophilic potential of HPβCD carrier was evaluated when encapsulated with suberoylanilide hydroxamic acid (SAHA), a potent histone deacetylase inhibitor enable to rescue lethality and photoreceptor neurodegeneration, in drinking water. HPβCD/SAHA complexes have demonstrated proven benefit in ameliorating motor impairment in preclinical trials with R6/2 mouse model [90]. In cell cultures expressing mutant *huntingtin*, treatments with either simvastatin or β-CD have demonstrated to reduce lipid content in cell membrane modifying in turn the membrane localization of *N*-methyl-d-aspartate (NMDA) receptors which are commonly associated with cholesterol-enriched domains in HD. Therefore, such effect rendered membrane NMDA receptors less accessible, protecting them against the NMDA-induced excitotoxicity [88].

## 6. Conclusions

We have discussed here a growing amount of evidence demonstrating that cyclodextrins represent an exciting tool to develop useful therapies based on their lipid-extracting action as well as to improve the efficacy of already existing therapies while included in novel drug formulation complexes. In the further development of CD-based therapy for individuals with cardiovascular and neurodegenerative diseases, a major key issue may be the unwanted side effects as reported for HPβCD in the cat model of NPC1, and that is actually in clinical trials for NPC1. Another point is by-passing the BBB to reach CNS affected structures in neurodegenerative disease. For example, treatment of NPC1 in humans would probably require different cumulative routes of access, even invasive, for fully effective treatment. Additionally, CDs can directly interact with the endothelial cells forming the BBB and modify cell membrane composition that directly influence cholesterol trafficking and homeostasis. Therefore, the effects of CDs on RCT still need to be fully addressed. Along with CD benefits observed in animal models, the critical question about the translation from animal models to human therapy is still raised.

## Figures and Tables

**Table 1:** Structure of native CDs and common modified CDs.

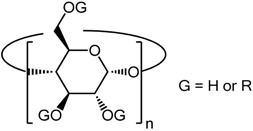			
Abbreviation	n	Substituent (R)	Number of R Group by CD
α-CD	6	(−)	0
β-CD	7	(−)	0
γ-CD	8	(−)	0
HPαCD	6	-CH_2_-CHOH-CH_3_	3.6
RAMEα	6	-CH_3_	10.8
HPβCD	7	-CH_2_-CHOH-CH_3_	5.6
KLEPTOSE^®^ CRYSMEB	7	-CH_3_	4
Methyl-β-CD	7	-CH_3_	1.6
RAMEβ	7	-CH_3_	12.6
SBE7-β-CD	7	-(CH_2_)_4_-SO_3_Na	7
TRIMETHYL-β-CD	7	-CH_3_	21
HPγCD	8	-CH_2_-CHOH-CH_3_	4.8
RAMEγ	8	-CH_3_	14.4

α-CD, α-cyclodextrin; β-CD, β-cyclodextrin; CD, cyclodextrin; CRYSMEB, crystalline methylated-β-cyclodextrin; γ-CD, γ-cyclodextrin; HPαCD, 2-hydroxypropyl-α-cyclodextrin; HPβCD, 2-hydroxypropyl-β-cyclodextrin; HPγCD, 2-hydroxypropyl-γ-cyclodextrin; Methyl-β-CD, methyl-β-cyclodextrin ; RAMEα, randomly-methylated-α-cyclodextrin; RAMEβ, randomly-methylated-β-cyclodextrin; RAMEγ, randomly-methylated-γ-cyclodextrin; SBE7-β-CD, sulfobutylether-7-β-cyclodextrin; TRIMETHYL-β-CD, TRIMETHYL-β-cyclodextrin.

**Table 2:** Summary of cyclodextrins (CDs) and their therapeutical uses in vascular and neurodegenerative diseases.

Disease	CD	Therapeutical Effect(s) of CDs	Reference
Atherosclerosis	β-CD	Cholesterol depletion	[12]
		Enhances cholesterol efflux from cell membranes	[52], alone or associated with chitosan and/or CEEP
		Selective binding to cholesterol crystals allowing early detection of plaques	[51], carboxy-methyl-β-CD conjugated with superparamagnetic ion oxyde nanoparticles
	RAMEβ	Cholesterol depletion	[12]
	KLEPTOSE^®^ CRYSMEβ	Reduced atherosclerotic plaque size in vivo and T cell content	[11]
		Cholesterol depletion	[11,12]
	Methyl-β-CD	Cholesterol depletion	[12]
	HPβCD	Cholesterol depletion	[13,53,54,55,56]
		Reduces inflammation and lowers atherosclerotic lesion formation, reduces amounts of cholesterol crystals in atherosclerotic plaques	[13]
Niemann–Pick type C	β-CD	Reduced intracellular cholesterol pool	[57], conjugated to octa-arginine
	HPβCD	Cholesterol depletion, reverses defective lysosomal transport, delays demyelination, recovers neuronal function, increased life expectation in animal models, reduces hepatic cholesterol level and liver dysfunction. Do not cross the mature BBB, ototoxicity	[54,58,59,60,61,62,63,64,65,66,67], associated with allopregnanolone
		Completed phase I clinical trial	[68], associated with itraconazole
Alzheimer’s disease	β-CD	Binds Aβ peptide, reduces Aβ neurotoxicity	[69,70,71]
		Nasal delivery system for brain targeting	[72], coupled with alginate or chitosan
		Nasal delivery system for brain targeting	[73], associated with tacrine/albumin
		Oral delivery system for brain targeting	[74], associated with chitosan and phospholipids
	HPβCD	Cholesterol depletion, improves spatial learning and memory deficits in animal model, decreases Aβ plaque deposition	[75]
		Nasal delivery system for brain targeting	[72], coupled with alginate or chitosan
		Nasal delivery system for brain targeting	[73], associated with tacrine/albumin
		Cryoprotective in drug delivery complex	[76], coupled with curcumin
		Improved brain delivery and neurogenic drug efficacy	[77], associated with allopregnanolone
		Nasal delivery system for brain targeting,	[78], coupled with chitosan or sodium alginate
	per-6-alkylamino-β-CD	Binds Aβ peptide, reduces Aβ neurotoxicity	[79]
	SBE7-β-CD	Nasal delivery system for brain targeting, decreases Aβ-induced neurotoxicity in rat hippocampus	[73], associated with tacrine/albumin
		Improved brain delivery and neurogenic drug efficacy	[77], coupled with allopregnanolone
Parkinson’s disease	β-CD	Nasal delivery system for l-DOPA into the brain	[80]
		Increases curcumin potency in inhibiting α-synuclein aggregation	[81], associated with curcumin
		Dissolves preformed α-synuclein aggregates	[82], associated with curcumin
	HPβCD	Activates the transcription factor EB, which is involved in the autophagy-lysosomal degradation pathway	[83]
		Nasal delivery system for l-DOPA into the brain	[84], coupled with L-DPOPA and maleic acid
		Enhances drug delivery across the BBB	[85], conjugated with dopamine receptor agonist
	methyl-β-CD	Reduces α-synuclein protein aggregation	[86]
	SBE7-β-CD	Nasal delivery system for l-DOPA into the brain	[87], incorporated in nanoparticles with l-DOPA
Huntington’s disease	β-CD	Reduces lipid content in cell membrane	[88]
		Enhances siRNA delivery to the brain	[89], coupled to htt siRNA
	HPβCD	Enhances drug delivery to the brain	[90], associated with SAHA

Aβ, β-amyloid peptide; AD, Alzheimer’s disease; β-CD, β-cyclodextrin; CD, cyclodextrin; BBB, blood-brain barrier; CEEP, cellular cholesterol efflux enhancing peptide; CRYSMEB, crystalline methylated-β-cyclodextrin; HPβCD, 2-hydroxypropyl-β-cyclodextrin; Htt, Huntingtin; Methyl-β-CD, methyl-β-cyclodextrin; per-6-alkylamino-β-CD, per-6-alkylamino-β-cyclodextrin; RAMEβ, randomly-methylated-β-cyclodextrin; SAHA, suberoylanilide hydroxamic acid; SBE7-β-CD, sulfobutylether-7-β-cyclodextrin; TRIMETHYL-β-CD, TRIMETHYL-β-cyclodextrin.
